# Mechanisms of hydrogen embrittlement, mitigation strategies, and emerging materials for hydrogen energy and advanced manufacturing

**DOI:** 10.3389/fchem.2026.1957974

**Published:** 2026-09-09

**Authors:** Pan Shulin, Walid Tahri, Rashid Khan, Mazen Alshaaer

**Affiliations:** 1 Key Laboratory of Yangtze Aquatic Environment, Research Base, Yibin University, Yibin, China; 2 Zhejiang Provincial Key Laboratory of Advanced Chemical Engineering Manufacture Technology, College of Chemical and Biological Engineering, Zhejiang University, Hangzhou, China; 3 Department of Physics, Prince Sattam Bin Abdulaziz University, Al-Kharj, Saudi Arabia

**Keywords:** hydrogen embrittlement, hydrogen transport, in situ characterization, metallic materials, multiscale modelling

## Abstract

Hydrogen is considered one of the most promising energy carriers for low-carbon energy systems. However, hydrogen embrittlement (HE) poses a threat to the structural integrity and long-term reliability of metallic materials used in hydrogen production, storage, transportation, and utilization. Microstructural features such as dislocations, vacancies, grain boundaries, precipitates, and phase interfaces can act as hydrogen trapping sites. Hydrogen atoms introduced through gaseous exposure, electrochemical reactions, or corrosion processes can accumulate at these sites. Consequently, hydrogen-metal interactions can reduce ductility, accelerate crack initiation and propagation, and promote premature failure under static and cyclic loading conditions. This brief overview discusses the recent advances in hydrogen uptake, diffusion, trapping, and damage mechanisms, including hydrogen-enhanced decohesion (HEDE), hydrogen-enhanced localized plasticity (HELP), and hydrogen-enhanced strain-induced vacancy formation (HESIV). HE susceptibility is examined in relation to alloy chemistry, microstructure, environmental factors, and mechanical loading conditions. In addition, recent advances in multiscale modelling, *in situ* and operando characterization techniques, artificial intelligence (AI)-assisted prediction, and hydrogen detection methodologies are discussed. Moreover, mitigation strategies involving microstructure optimization, surface engineering, protective coatings, heat treatment of materials, and stress management are investigated. This review compares and contrasts promising emerging materials for hydrogen energy and advanced manufacturing, such as high-entropy alloys, additively manufactured alloys, and advanced high-strength steels, in contrast to other reviews that primarily focus on specific alloy types or HE mechanisms. Finally, future research priorities are identified, including standardized evaluation procedures, testing under real-world service conditions, data standardization, model validation, and the development of safe, durable, hydrogen-compatible metallic materials.

## 1 Introduction

To minimize the environmental impact associated with transportation, manufacturing, energy storage, and electricity generation, hydrogen is gaining popularity as a potential energy carrier. However, the widespread adoption of hydrogen technologies requires a robust infrastructure and technological framework for its production, compression, storage, transportation, and utilization. Metallic materials are extensively employed in pipelines, storage tanks, compressors, valves, electrolyzers, fuel cells, and offshore systems due to their high mechanical strength, manufacturing, and cost-effectiveness. Nevertheless, exposure to hydrogen gas, electrochemically generated hydrogen, or hydrogen released during corrosion reaction can significantly degrade the mechanical properties of these materials. Consequently, HE poses a critical challenge to the safety, long-term reliability, and structural integrity of hydrogen energy infrastructure (Liu P.-Y. et al., 2024; Pallaspuro et al., 2024).

HE typically occurs via a series of interconnected processes, including hydrogen generation and surface reactions, diffusion, trapping, and stress-assisted accumulation within the metal matrix (Cupertino-Malheiros et al., 2024; Sun et al., 2025). Hydrogen atoms can be introduced into metals through various pathways, such as cathodic polarization, electroplating, welding operations, aqueous corrosion, or contact with high-pressure hydrogen gas (Cupertino-Malheiros et al., 2024; Sun et al., 2025; Hopf et al., 2024; Williams et al., 2024). Once absorbed, hydrogen diffuses through the lattice and interact with microstructural features, including voids, dislocations, grain boundaries, inclusions, precipitates, and phase interfaces (Islam et al., 2025; Liu M. et al., 2024). These microscopic characteristics can either facilitate hydrogen transport or act as irreversible/reversible trapping sites, promoting localized hydrogen accumulation in the regions susceptible to mechanical degradation. Recent studies have demonstrated that hydrogen diffusivity and trapping behavior in pipeline steels are extremely sensitive to grain size, pearlite fraction, dislocation density, and prior thermomechanical processing history. Specifically, modern pipeline steel has reduced hydrogen permeability compared to conventional pipeline steel, which suggests that microstructure plays a direct role in controlling hydrogen transport and potential embrittlement susceptibility (Islam et al., 2025).

Mechanical degradation can be caused by even modest amounts of diffusible hydrogen, particularly when hydrogen exposure coincides with applied residual stresses. Consequently, common consequences include reduced elongation, lower fracture toughness, accelerated fatigue-crack growth, delayed fracture, and transitions from ductile fracture to quasi-cleavage or intergranular cracking (Ghadiani et al., 2025; Konert et al., 2025; Myhre et al., 2025; Wei et al., 2024). For instance, *in situ* hydrogen charging of ultra-high-strength R6 mooring chain steel caused a 38.94% reduction in elongation while producing intergranular cracking above a critical cathodic charging potential (Wei et al., 2024). A similar study by Konert et al. (2025) showed that when X65 pipeline steel was subjected to slow-strain-rate testing in 60 bar gaseous hydrogen, the area at fracture decreased from 72% to 52% in cross-weld specimens and from 73% to 51% in base-metal specimens accompanied by multiple cracking mechanism and cleavage features.

Additionally, Ghadiani et al. (2025) demonstrated through double-cantilever-beam (DCB) studies that a mere 0.5 wppm hydrogen decreased the fracture toughness of contemporary X65 steel by 14.1% and in retrograde X52 steel by 5.5%. Similarly, a study conducted by Myhre et al. (2025) found that cyclic loading accelerated fatigue-crack growth by up to one order of magnitude in both vintage and modern X65 pipeline steels, with a higher acceleration associated with an increased fraction of quasi-cleavage fracture. Moreover, plastic deformation can further increase hydrogen uptake by generating additional dislocations and vacancies that act as hydrogen-trapping sites. In this regard, experimental work on ferritic high-strength steel confirmed that increasing plastic strain during hydrogen charging increased the absorbed hydrogen concentration and intensified hydrogen-related degradation (Boot et al., 2025a).

Hydrogen-induced mechanical degradation is governed by multiple interacting mechanisms rather than a single dominant process. Instead, hydrogen-enhanced localized plasticity, hydrogen-enhanced decohesion, vacancy-mediated damage, adsorption-induced dislocation emission, and hydride formation can operate individually or simultaneously, depending on the alloy, microstructure characteristics, hydrogen concentration, stress state, strain rate, and temperature. For example, direct *in situ* studies in α-iron have shown that hydrogen reduced, the critical stress required to initiate screw-dislocation motion was reduced by 27%–43%, providing quantitative evidence that hydrogen enhances dislocation mobility (Huang et al., 2023). Conversely, recent *in situ* tensile observations of Ni-based alloy 725 showed that hydrogen-assisted cracking is not exclusively associated with localized plastic deformation, as crack initiation was also observed at microstructural stress concentrators such as inclusions, triple junctions, and surface defects without detectable slip activity.

Moreover, recent studies on martensitic steel have shown that hydrogen infiltration can be significantly reduced, and resistance to hydrogen-assisted interfacial cracking can be enhanced by enriching the material with boron and carbon to avoid hydrogen segregation at susceptible interfaces (Hachet et al., 2025). Collectively, these results suggest that hydrogen-induced cracking mechanisms are highly dependent on the local microstructural environment: in some cases, cracking may be promoted by localized plasticity, whereas in others, interfacial decohesion or defect-controlled fracture mechanisms may become dominant (Liu M. et al., 2024). Recent studies therefore treat microstructural defects as designable features rather than unavoidable causes of degradation. To increase hydrogen-trapping capacity of steel, Liu P.-Y. et al. (2024) developed Ti-Mo carbide precipitates containing carbon vacancies, which served as effective hydrogen traps. However, hydrogen resistance is governed not only by trap density but also by the intrinsic characteristics of trapping sites. Whether a microstructural feature safely immobilizes hydrogen or concentrates it at a fracture-sensitive region depends on factors including size, coherency, reversibility, trap capacity, and spatial position (Boot et al., 2025b; Hammer et al., 2024; Safyari et al., 2024). To illustrate, Boot et al. (2025b), revealed that coherent vanadium carbides offered an optimal combination of strength, ductility, and hydrogen resistance. While, larger semi-coherent titanium carbides were found to increase intergranular fracture and encourage hydrogen accumulation near grain boundaries. Another way to manage the distribution of hydrogen is by retained-austenite morphology. HE resistance of 2 GPa direct-quenched and partitioned martensitic steels was enhanced by thicker film-like retained austenite and aluminum alloying, as demonstrated by Pallaspuro et al. (2024). According to these findings, hydrogen trapping is effective only when it decreases mobile hydrogen without causing locally brittle areas.

Furthermore, microstructure homogenization and post-processing have also contributed to advancements. For example, heat treatment of 316L stainless steel that had been directed-energy deposited resulted in a more homogenous microstructure, decreased HE sensitivity to levels seen in conventionally manufactured 316L stainless steel, and reduced subgrain structures and dislocation density (Liu Q. et al., 2025). Moreover, while CO_2_ accelerated the formation of hydrogen-assisted fatigue cracks in L360 pipeline steel under high-pressure hydrogen, small amounts of O_2_ prevented HE and partially offset CO_2_’s effect (Zhou et al., 2024a). Nevertheless, laboratory results remain difficult to transfer directly to operating components because hydrogen pressure, electrochemical potential, temperature, loading rate, weld condition, residual stress, and environmental impurities vary widely among hydrogen technologies.

The links among microstructure, hydrogen exposure, mechanical loading, and fracture behavior are the main emphasis of this brief review, which also outlines recent advances in understanding the mechanisms of hydrogen uptake, transport, entrapment, and hydrogen-assisted damage in metallic materials. New developments in mitigation measures, such as *in situ* analysis, computation-based prediction, alloy design, microstructural engineering, surface protection, and hydrogen characterization, are also highlighted. Finally, key research challenges are identified to guide materials innovation and support the development and qualification of hydrogen-compatible materials for sustainable energy and industrial applications.

HE has been the subject of multiple recent assessments, each of which takes a somewhat different but complementary approach. A literature review was conducted by Rajput, Arya, and Jurwall et al. (2026), on HE mechanisms, testing methodologies, and mitigation in automotive high-strength steels. Meanwhile, surface-modification tactics were the focus of Maurya and Akhtar (2026), whereas HE in gaseous hydrogen pipes was investigated by Cheng et al. (2026). In contrast, this review offers four more comprehensive contributions: (i) a framework for comparing HELP, HEDE, vacancy-mediated damage, and hydride formation, with evidence, vulnerable materials, and charging conditions linked to each mechanism (Section 2.3; Table 1); (ii) a quantitative comparison across alloys, including ferritic/martensitic and austenitic steels, Ni-based, Ti, and Al alloys, under different hydrogen sources and loading modes (Table 2); (iii) an integrated assessment that links characterization and modeling approaches to mitigation strategies, including their advantages, disadvantages, scalability, and service-condition constraints (Sections 4–5); and (iv) a consistent focus on the gap between controlled laboratory studies and complex, multi-variable service conditions.

**TABLE 1:** Analysis and comparison of the main processes that lead to metallic materials being damaged by hydrogen.

Mechanism	Proposed driving force	Characteristic microstructural evidence	Typical susceptible materials	Relevant H-charging conditions	Fracture morphology	Refs.
HELP (hydrogen-enhanced localized plasticity)	Hydrogen can cause dislocation motion, slip localization, or heterogeneous dislocation accumulation. It can soften or harden locally depending on hydrogen content, dislocation character, and strain rate	Localized or flat slip bands around the fracture tip; heterogeneous dislocation structures; *in situ* microscopy or diffraction-observed hydrogen-associated dislocation unpinning, mobility, or shielding	Austenitic stainless steels, BCC steels, FCC nickel and nickel-based alloys, and any alloys where hydrogen interacts strongly with movable dislocations	Deformation necessitates that mobile hydrogen reaches active dislocations. When the temperature, hydrogen diffusivity, applied strain, and strain rate are all optimal, HELP becomes the dominant process	Intense slip bands, ductile striations, or small, localized slip marks; HELP usually works in combination with other processes	Sun et al. (2023), Yang et al. (2025)
HEDE (hydrogen-enhanced decohesion)	When there’s a high local tensile stress and enough hydrogen coverage, the cohesive strength of the material decreases because hydrogen tends to settle at grain boundaries, phase boundaries, or cleavage planes	Fracture routes linked to chemically weakened or hydrogen-enriched borders; deuterium or hydrogen enrichment at grain boundaries or interfaces, as identified by APT or similar nanoscale techniques	Materials having interfaces that strongly trap hydrogen, such as high-Mn steels, Ni-based alloys, and steels with segregation-prone prior-austenite-grain boundaries	Promote local hydrogen accumulation without requiring total segregation equilibrium with sustained hydrogen exposure and sufficient diffusion time, static or slowly increasing load, and high hydrostatic tensile stress	Local plastic deformation may still happen close to the crack tip, but macroscopic plasticity is limited; most fractures are intergranular or cleavage-like	Azócar Guzmán and Janisch (2024), Khanchandani and Gault (2023)
Vacancy-mediated (hydrogen-enhanced strain-induced vacancy) damage (HESIV)	For vacancies created by plastic deformation, hydrogen acts as a stabilizer, inhibits vacancy annihilation, and encourages their aggregation and clustering into nanovoids	Clustering of vacancies and development of nanovoids in extremely stressed areas; increased concentration of vacancy-type defects as identified by positron annihilation spectroscopy or low-temperature thermal desorption	Evidenced in a variety of severely deformed hydrogen-containing alloys, including BCC iron and ferritic steels, FCC austenitic stainless steels, and others	Concurrent hydrogen exposure and substantial plastic strain are generally required. Temperature and deformation rate control vacancy generation, migration, and annihilation	Rapid nucleation, development, and coalescence of micro-voids within a ductile-looking fracture that often overlaps with HELP or HEDE characteristics	Komatsu et al. (2021), Sugiyama and Takai (2021)
Hydride formation	Brittle hydrides form when the concentration of hydrogen in a certain area grows too high for it to remain a solid at room temperature. Their position, elastic mismatch, transformation strain, and contact with slip all work together to cause cracks to start and spread	Lattice strain surrounding hydrides; slip localization and microcracking inside or close to hydrides; XRD, EBSD, TEM, and SEM detection of hydride platelets or needles	Mostly alloys and metals that can create hydrides, such as Zr, Ti, and a few HCP alloys. Under typical operating circumstances, this is not a mechanism that steels or Ni alloys typically exhibit	Conditions that can lead to hydrogen redistribution and hydride reorientation include a high local hydrogen concentration; enough diffusion or cooling time to achieve supersaturation; extended gaseous or electrochemical charging; and stress	Hydrides or the interfaces between hydrides and matrix materials can start cracking. Several factors, including hydride orientation, shape, spacing, and applied stress, determine whether crack pathways are intergranular or trans granular	Chen et al. (2025), Liu et al. (2023), Marashi and Abdolvand (2024)

The listed responses are major tendencies. Actual susceptibility depends on composition, processing, hydrogen source, concentration, temperature, pressure, stress state, and exposure duration; with BCC: body-centered cubic, and FCC: faced centered cubic. XRD: x-ray Diffraction, EBSD: electron backscatter diffraction, TEM: transmission Electron Microscopy, SEM: scanning electron microscopy, and HCP: hexagonal close-packed alloys.

**TABLE 2:** Comparing hydrogen exposure, composition, and mechanical and mechanistic reactions in metallic materials across studies.

Alloy/material	Hydrogen source	Pressure/charging current	Temperature	Loading mode	Measured H content	Mechanical response	Principal mechanism reported	Ref.
R6 ultra-high-strength mooring-chain steel	Electrochemical (cathodic)	Above a critical cathodic potential	Ambient	*In-situ* hydrogen charging, tensile	Not separately quantified in source	38.9% reduction in elongation; intergranular cracking above critical potential	HEDE (intergranular)	Wei et al. (2024)
X65 pipeline steel (base metal/cross-weld)	Gaseous H2	60 bars	Ambient	Slow strain-rate testing	Not quantified in source	Area-at-fracture fell 72%→52% (cross-weld), 73%→51% (base metal)	Mixed cracking/cleavage	Konert et al. (2025)
X65 steel (contemporary)/X52 steel (retrograde)	Not specified (DCB testing)	Not specified	Ambient	Double-cantilever-beam fracture-toughness	0.5 wppm	Fracture toughness reduced 14.1% (X65) and 5.5% (X52)	Decohesion-controlled fracture toughness loss	Ghadiani et al. (2025)
Vintage vs. modern X65 pipeline steel	Gaseous/electrochemical (permeation)	Not specified	Ambient	Cyclic loading (fatigue)/permeation	Vintage: 50% higher permeation current, 97% higher effective diffusivity	Fatigue-crack growth accelerated up to 1 order of magnitude; more acceleration with quasi-cleavage fraction	Microstructure-controlled transport; mixed mechanisms	Islam et al. (2025)
Ferritic high-strength steel	Electrochemical charging during plastic straining	Not specified	Ambient	Tensile with concurrent plastic strain	2.36→3.69 wppm (0%→3% plastic strain)	Increased hydrogen uptake with plastic strain; intensified degradation	Strain-induced trap generation (dislocations)	Myhre et al. (2025)
Ti-6Al-4V	Electrochemical (current-controlled)	Controlled charging current	Ambient	Tensile	Not quantified (hydride vs. solute H separated)	32.85% decrease in elongation; hydride development exacerbated intergranular cracking	Hydride formation	Boot et al. (2025a)
X80 pipeline steel	Hydrogen-blended natural gas (with corrosion-inducing impurities)	8 MPa	Ambient	Tensile	Not separately quantified	Elongation fell to 14.3% (below 18% API 5L X80 requirement); fewer/smaller dimples, more quasi-cleavage	Mixed HELP/HEDE with corrosion-impurity interaction	Wang et al. (2025)

## 2 Hydrogen uptake, transport, trapping and damage

### 2.1 Hydrogen uptake and transport

HE begins with the generation or dissociation of hydrogen at a metal surface. In aqueous environments, atomic hydrogen can form through corrosion, cathodic protection, electroplating, pickling, or electrochemical hydrogen evolution. The rate-controlling surface reaction, surface activity, electrolyte pH, and cathodic overpotential are the most important factors in hydrogen evolution and absorption, according to electrochemical impedance spectroscopy, polarization studies, and permeation testing on iron and nickel (Cupertino-Malheiros et al., 2024). Nevertheless, there is still a lack of adequate quantification of the relative importance of each surface attribute under real service conditions.

In contrast, under gaseous exposure, molecular hydrogen first adsorbs and dissociates before atomic hydrogen enters the metal. The presence of oxygen and water impurities in Fe (100) has been shown to significantly affect hydrogen adsorption and dissociation. Specifically, these impurities compete for surface sites, modify surface charge, and change the energy barrier for hydrogen dissociation, according to recent results from density functional theory and first-principles molecular dynamics calculations (Xu et al., 2025). Therefore, surface oxides, contaminants, electrolyte composition, electrochemical potential, hydrogen pressure, and temperature determine the amount of hydrogen available for absorption. In some cases, surface oxides are more than just a permeation barrier. For example, pre-existing oxides enhanced total hydrogen uptake during combustion exposure and inhibited subsequent hydrogen desorption, according to flame-charging studies conducted on nickel-base superalloys (Schu et al., 2025). These results, however, have limited generalizability due to their reliance on oxide composition, thickness, and exposure conditions.

The kinetics of hydrogen entry differ among the four main hydrogen sources investigated in this review. During electrochemical charging, atomic hydrogen is generated at the metal surface, and hydrogen uptake depends strongly on electrolyte chemistry, the applied potential or current density, and the presence of recombination poisons (Hagen et al., 2024; Koren et al., 2023; Koren et al., 2024). Aggressive cathodic charging can produce subsurface hydrogen concentrations comparable to, or even higher than, those produced by high-pressure gaseous exposure. Therefore, these conditions should be compared using the resulting subsurface hydrogen concentration rather than the nominal charging parameters.

Gaseous hydrogen entry requires molecular adsorption, dissociation, and subsequent absorption. These processes depend on hydrogen partial pressure, surface conditions, corrosion-product films, and competing gas species (Koren et al., 2023; Xu et al., 2024). For example, CO and CO_2_ can decrease hydrogen entry in hydrogen-blended natural gas by limiting surface adsorption and dissociation (Zhang R. et al., 2024). Consequently, embrittlement severity should be related to hydrogen partial pressure, total pressure, and complete gas composition rather than blend percentage alone. Moreover, compact FeCO_3_ films can reduce hydrogen permeation, whereas defects in these films can create localized hydrogen-entry sites (Xu et al., 2024). Therefore, quantitative comparisons among different exposure routes should report or estimate the subsurface or diffusible hydrogen concentration rather than relying solely on nominal pressure, blend percentage, potential, or current density.

Once absorbed, hydrogen occupies interstitial lattice sites and moves under concentration, stress, temperature, and chemical-potential gradients. At dislocations, vacancies, grain boundaries, precipitates, inclusions, and phase interfaces, its mobility is affected by both reversible and irreversible trapping. Thus, current coupled transport models encompass a variety of processes, such as lattice diffusion, stress-assisted migration, evolution of dislocation trap density, local electrochemistry, and hydrogen transport linked to dislocation motion, among others (Díaz et al., 2025). However, many models rely on uncertain transport parameters and oversimplified trap distributions.

Likewise, hydrogen transport differs considerably among crystal structures. Compared to body-centered cubic (BCC) ferrite, face-centered cubic austenite has a far lower hydrogen diffusivity and higher hydrogen solubility, according to experimental data that combined cyclic voltammetry (CV) with cryogenic-transfer atom probe tomography (APT) (Barton et al., 2024). Furthermore, hydrogen flux was higher through BCC ferrite than through face-centered cubic (CFC) austenite in UNS S32750 super-duplex stainless steel when imaging directly using a microscope. The size of the phase grains and the distance from the ferrite-austenite boundary affected the amount and location of the hydrogen flux as it migrated from ferrite to austenite (Kakinuma et al., 2024). Despite these advances, it remains difficult to compare directly the quantitative results of various characterization methods.

Moreover, in multiphase materials, each phase may therefore act as a transport pathway, temporary reservoir, or barrier. For example, Islam et al. (2025) compared modern, vintage, and legacy natural-gas pipeline steels and found that hydrogen transport increased with grain size and pearlite content but decreased with dislocation density. For instance, vintage steel displayed 50% higher steady-state permeation current and 97% greater effective diffusivity than modern steel. The finer grains and higher dislocation density of the modern steel delayed hydrogen permeation, showing that bulk alloy composition alone cannot predict hydrogen transport. In addition, applied stress further modifies hydrogen distribution. Hydrostatic tensile stress decreases the chemical potential of hydrogen and promotes its movement toward crack tips, notches, phase boundaries, and other highly stressed regions (Li et al., 2024). Moreover, plastic deformation creates new dislocations, vacancies, and interfaces that provide additional transport paths and trapping sites. Most of the extra trapped hydrogen in ferritic high-strength steel came from dislocations; however, the amount of hydrogen absorbed rose from 2.36 to 3.69 wppm when plastic strain was increased from 0% to 3%. However, it remains difficult to experimentally separate the impacts of phase fraction, applied stress, grain size, and dislocation density.

Consequently, hydrogen concentration near an advancing crack may differ substantially from the average concentration measured in the bulk material. In addition, weld areas and X52 pipeline steel were recently modeled with microstructure-resolved computational methods. These methods effectively combined chemical-potential-based hydrogen diffusion with crystal plasticity, and they were able to replicate fatigue trends found experimentally at both macroscopic and microscopic scales (Muth et al., 2025). Hydrogen uptake, deformation, and crack propagation should therefore be treated as coupled processes rather than independent stages.

Additional testing under realistic hydrogen environments and variable loading conditions is therefore necessary.

### 2.2 Hydrogen trapping at microstructural defects

Hydrogen traps are microstructural sites where hydrogen has a lower energy than at regular lattice positions. Specifically, common traps include vacancies, dislocations, grain boundaries, inclusions, precipitates, interfaces, and micro-voids. Traps with relatively low binding energies release hydrogen readily and are often termed reversible traps. In contrast, stronger traps retain hydrogen for longer periods and are commonly described as irreversible under a defined experimental temperature and timescale. For instance, a recent study of ferritic Fe-Cr alloys has shown that hydrogen uptake is enhanced by dislocations, grain boundaries, and substitutional Cr atoms, but its apparent diffusion coefficient is reduced. In a similar manner, research on ferritic steels that contain carbides has also shown that interstitial sites at carbide-matrix interfaces and misfit-dislocation cores have distinct trapping strengths (Hammer et al., 2024; Rao et al., 2025). However, because factors such as trap-binding energy, temperature, exposure time, local stress, diffusion distance, and the presence of a linked transport pathway affect hydrogen release, the reversible or irreversible categorization is a practical one.

Hydrogen trapping impacts multiple partly independent transport and damage characteristics; thus, enhancing one does not inevitably increase hydrogen resistance. Lattice diffusivity describes hydrogen mobility through a nominally defect-free crystal lattice, while effective, or apparent, diffusivity is the macroscopically measured, trap-retarded value that depends on trap density, occupancy, and experimental timescale (Cupertino-Malheiros et al., 2023). Trap density dictates trapping sites, while trap-binding energy affects hydrogen retention and release kinetics. Practically, reversible and irreversible trapping depend on temperature and timing, not material qualities (Cupertino-Malheiros et al., 2023; Komatsu et al., 2021). Vacancies and deformation-induced lattice defects can store hydrogen, coupling trapping to plastic deformation and vacancy accumulation (Komatsu et al., 2021; Sugiyama and Takai, 2021).

The mechanical effect varies on the local hydrogen concentration at a fracture tip, grain boundary, or phase contact compared to the damage threshold. Local stress can shift hydrogen toward grain boundaries, and significant hydrogen coverage can weaken grain boundaries (Azócar Guzmán and Janisch, 2024). Well-distributed, stable traps may minimize hydrogen availability at crack-sensitive regions, improving resistance. Traps near mechanically sensitive contacts may increase hydrogen accumulation and breakage (Safyari et al., 2024). Reduced effective diffusivity alone does not increase hydrogen resistance unless hydrogen is kept away from cracks”.

Boot et al. (2025b) found that coherent VC nanoparticles provided the best combination of strength, ductility, and hydrogen resistance. In contrast, larger semi-coherent TiC precipitates near grain boundaries accelerated intergranular fracture and concentrated hydrogen within adjacent dislocation pile-ups. Similarly, evidence of comparable advantages has been found that aluminum alloys with a high concentration of intragranular Al_3_(Mg,Sc)_2_/Al_3_Sc core-shell nanoprecipitates exhibited approximately five times greater HE resistance than the Sc-free alloy (Jiang et al., 2025). These findings emphasize the importance of precipitate position and coherency, rather than precipitate density alone.

Nevertheless, the same microstructural feature may simultaneously trap hydrogen and promote damage. For example, Santos Maldonado et al. (2024) compared wrought and additively manufactured Inconel 718. The additively manufactured alloy contained dense dislocation-cell walls and Laves phases that trapped hydrogen. However, the cellular dislocation network also accelerated hydrogen penetration and concentrated hydrogen along the cell walls, causing strength loss and intragranular cracking. The wrought alloy, which lacked this extensive cellular structure, showed mainly intergranular fracture. In this regard, recent research studies in interface engineering using martensitic steel support this result. As reported by Hachet et al. (2025), when carbon and boron were segregated at grain boundaries that were sensitive to hydrogen, it prevented hydrogen from segregating, cut hydrogen ingress in half, and made the material much more resistant to interfacial cracking caused by hydrogen. Microstructural design must thus prioritize the isolation of hydrogen from mechanically weak borders and interfaces and its retention at stable intragranular locations. Indeed, additional research is needed to determine how well these designed traps and interfaces hold up under cyclic stress and extended exposure to hydrogen.

### 2.3 Hydrogen-assisted damage mechanisms

New experimental studies show that there are several mechanisms that control hydrogen-assisted mechanical failure; the relative importance of these mechanisms change with material composition, microstructure, hydrogen concentration, loading mode, strain rate, temperature, and crack-tip stress state (Liu M. et al., 2024; Chiari et al., 2025; Kim et al., 2025; Shi et al., 2025).

First, HELP, which stands for hydrogen-enhanced localized plasticity, is the theory that hydrogen helps dislocations move and focuses plastic deformation in small areas close to crack tips or microstructural flaws. New direct findings corroborate a complicated relationship between hydrogen and dislocations. To illustrate, Kim et al. (2025) investigated ferritic stainless steel using molecular dynamics modeling and *in situ* electron channeling contrast imaging. At first, dislocation movement was caused by hydrogen flux alone, without any external load. Pinning of the dislocations occurred when hydrogen built up in their centers. Thus, hydrogen can either increase mobility or decrease it depending on the dislocation arrangement and local concentration of hydrogen.

Nevertheless, not all hydrogen-assisted fracture initiation may be explained by localized slip. For example, testing of hydrogen-exposed Ni-based alloy 725 was conducted by Liu M. et al. (2024) using *in situ* tensile testing. Some cracks started close to strong slip bands, which suggests that localized plasticity played a role. However, cracks also developed at inclusions, triple junctions, and surface defects without evident prior slip activity. Therefore, although not always required, the findings indicated that slip can facilitate crack initiation. Overall, the interaction between local plasticity, interfacial weakening, and microstructural stress concentration is supported by this study.

Second, according to the hydrogen-enhanced decohesion theory (HEDE), atomic bonding at grain boundaries, phase interfaces, and cleavage planes weakens due to hydrogen. Cracking can occur at stresses lower than those needed for hydrogen-free material due to hydrogen segregation, which reduces the resistance of these surfaces to separation. Likewise, because intergranular cracking is sensitive to grain-boundary chemistry, this theory has strong experimental backing. For instance, Hachet et al. (2025) showed that the segregation of boron and carbon decreased the buildup of hydrogen at susceptible interfaces by combining atomistic calculations with experiments conducted on martensitic steel. Hydrogen ingress was reduced by approximately half, resistance to hydrogen-assisted fracture was considerably boosted by the customized surfaces. Thus, to reduce damage caused by HEDE, the results show that competitive segregation and interfacial cohesiveness are important.

Third, vacancies created by plastic deformation can be stabilized and clustered, and micro-voids can be formed more quickly using hydrogen, according to the theory of hydrogen-enhanced strain-induced vacancy production. Under conditions of significant plastic strain, this process may slow down the cracking process and decrease ductility. However, voids, dislocation activity, and interface commonly develop together. Therefore, maintaining a clear distinction between HELP and HEDE and vacancy-mediated damage is challenging. Based on these results, vacancy accumulation is not a separate mechanism but instead is a component of a network of interrelated processes involving hydrogen-assisted damage.

Finally, one further clear way that hydride-forming metals, especially zirconium and titanium alloys, degrade is by hydride creation. The precipitation of brittle hydride phases occurs when the local hydrogen concentration exceeds the solid-solubility limit. These precipitates expand the lattice, generate internal stresses, cause local hardening, and create preferential crack-initiation sites. For example, Nguyen et al. (2024) used electrochemical charging current control to differentiate between the effects of solute hydrogen and hydride production in Ti6Al4V. A 32.85% decrease in overall elongation was due to the substantial hydride development, which exacerbated intergranular cracking primarily but also provided alternative crack-initiation and propagation pathways. According to Table 1, the driving forces, characteristic evidence, susceptible materials, hydrogen-charging conditions, and fracture features with the four main HE mechanisms depend on the material, microstructure, hydrogen availability, loading conditions, and the relative contribution of the interacting mechanisms.

It is possible for all of these processes to be active at once, as shown in Table 2. When hydrogen segregation at interfaces occurs in conjunction with intergranular fracture, HEDE is supported (Khanchandani and Gault, 2023; Sun et al., 2023), whereas HELP is characterized by hydrogen-modulated dislocation activity. Nonetheless, HELP is not necessarily necessary because fracture initiation in Alloy 725 can occur without prior localized slip (Liu et al., 2023). Sugiyama and Takai (Sugiyama and Takai, 2021), found that HESIV, in conjunction with HELP and HEDE, probably participates by accumulating vacancies and forming nanovoids. A small number of metals, including Ti and Zr alloys, are known to undergo hydride-assisted cracking (Liu et al., 2023; Marashi and Abdolvand, 2024).

**FIGURE 1:**
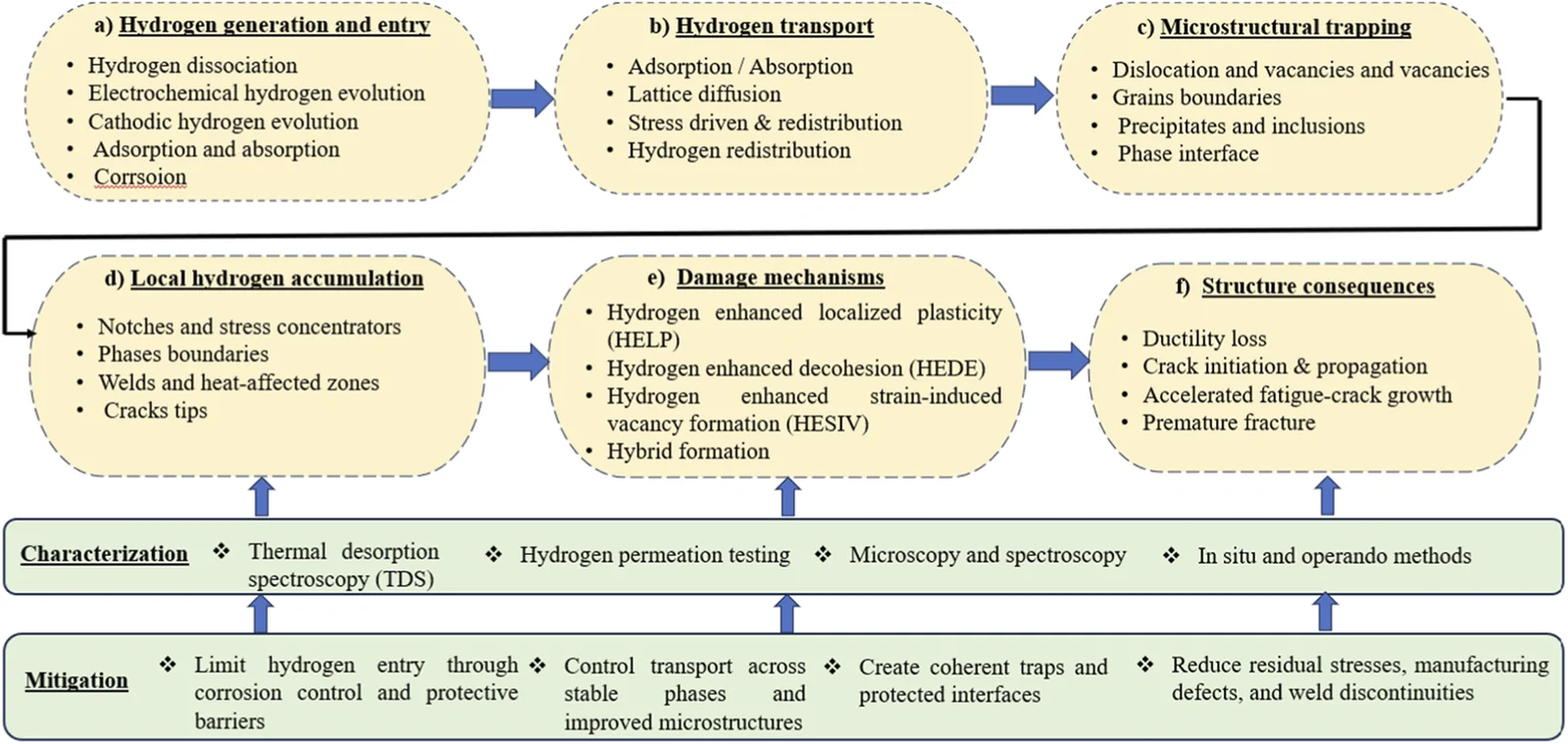
Interconnected pathway of hydrogen-assisted degradation, characterization, and mitigation in metallic materials.

Overall, HE involves surface and dissociation, followed by hydrogen entry and diffusion, trapping, stress-assisted accumulation, crack initiation, and failure. These processes span multiple scales and require integrated characterization, modelling, and mitigation approaches. The hydrogen-assisted degradation pathway is shown in Figure 1. It is divided into four stages: entry, transport, trapping, and damage. The methodologies used to characterize and evaluate these processes, as well as mitigation options, are detailed at different points in the pathway.

According to Figure 1. The characterization and mitigation of hydrogen-assisted deterioration along an intercommunicating pathway. The model differentiates between the following: input and production of hydrogen, transportation, microstructural trapping, local accumulation, processes of damage, and structural outcomes. It also details the characterization techniques that have been employed to discover these processes, as well as mitigation tactics that might be implemented at various points.

Interactions between surface, transport, trapping, deformation, and fracture processes cause HE. Hydrogen transport and redistribution are controlled by crystal structure and microstructure, while surface reactions control hydrogen entrance. Microstructural flaws capture hydrogen, whereas stress and plastic deformation create more trapping sites and local accumulation at damage-prone regions. These interactions can cause localized plasticity, interfacial decohesion, vacancy creation, or hydride formation. Thus, testing and modeling under application-relevant hydrogen exposure, temperature, pressure, and stress conditions are needed to determine the primary mechanism and estimate material susceptibility.

## 3 Material and environmental factors controlling hydrogen embrittlement

Mechanical loads, hydrogen availability, microstructure, and material chemistry combine to determine HE vulnerability. Due to hydrogen’s non-uniform distribution, materials with comparable bulk hydrogen concentrations may exhibit varying fracture reactions. It tends to condense in places that are microstructurally flawed or extremely strained. Hence, the amount of hydrogen in a material is not a good indicator of how well it will function.

Microstructure of the alloy, hydrogen source, charging circumstances, concentration of hydrogen, and loading mode are the variables that influence hydrogen-induced mechanical degradation, according to recent experimental investigations. Hence, it is not possible to infer better hydrogen resistance from decreased hydrogen diffusivity or enhanced trapping on their own. New evidence has been found to connect the dots between hydrogen uptake and transport, the mechanical response that follows, and the embrittlement mechanisms that have been postulated (Table 2).

Collectively, these investigations demonstrate that mechanical degradation is not directly proportional to bulk hydrogen content or effective diffusivity. The distribution of hydrogen among various areas in the structure, such as lattice sites, dislocations, grain boundaries, hydrides, and crack-tip regions, dictates the mechanical implications. Therefore, when assessing hydrogen resistance, it is necessary to take into account both the movement of hydrogen and its properties in the trap as well as its local accumulation, microstructure, stress level, and loading history.

### 3.1 Alloy composition and microstructure

While the susceptibility of a material to HE is significantly impacted by its strength, it is not possible to estimate performance just by strength without also taking microstructural features into account. For example, a higher hydrogen susceptibility and varied fracture behavior were observed in studies of modified AISI 4130 steel with varying degrees of strength (Martiniano et al., 2025). Therefore, greater local stresses and less plastic stress redistribution cause higher-strength materials to have lower tolerance to hydrogen-assisted cracking. Thus, mechanical strength should be assessed alongside other criteria such as grain-boundary properties, inclusions, dislocation density, phase distribution, grain size. As an illustration, recent pipeline-steel studies showed that processing history and microstructure alter hydrogen-transport performance in similar bulk compositions. Vintage pipeline steel has 50% higher steady-state permeation current and 97% higher effective diffusivity. Large grain size and pearlite content increased hydrogen diffusion, while high dislocation density decreased it. Overall, microstructural characteristics matter more than alloy composition and nominal strength for hydrogen susceptibility (Islam et al., 2025). The main metallic materials utilized in hydrogen systems are examined in Table 3 according to their hydrogen-transport characteristics, principal damage hazards, and priority mitigation techniques.

**TABLE 3:** Comparative hydrogen response and mitigation priorities for major metallic materials used in hydrogen systems.

Material class	Hydrogen transport and trapping	Main damage risks	Priority mitigation	Ref
High-strength ferritic and martensitic steels	Relatively rapid diffusion through the body-centered cubic (BCC) structure. Hydrogen can be trapped at dislocations, vacancies, carbides, inclusions, and prior-austenite grain boundaries. Trap effects depend on binding energy, reversibility, density, and spatial distribution	Intergranular or quasi-cleavage fracture, delayed cracking, and fatigue acceleration	Introduce finely dispersed, stable trapping sites, such as selected coherent or semi-coherent carbides; control retained-austenite stability; reduce inclusions and segregations; optimize interfaces; and minimize welding-induced residual stress	Boot et al. (2024), Boot et al. (2025b), Liu P.-Y. et al. (2024), Pallaspuro et al. (2024), Peng et al. (2024)
Austenitic stainless steels	Higher hydrogen solubility but generally slower lattice diffusion through the face-centered cubic (FCC) structure than in body- centered cubic (BCC) steels hydrogen transport and localization can increase along defects, higly strained regions, and strain-induced martensite	Ductility loss, secondary cracking, fatigue damage, and localization near transformed or highly strained regions	Stabilize austenite phase; suppress detrimental strain-induced martensitic transformation; control grain size, segregation, and inclusions; reduce residential stress; and limit hydrogen entry through surface engineering	Cho et al. (2023), Liu et al. (2025a), Yun et al. (2024)
Nickel-based alloys	Generally slow bulk hydrogen diffusion, but enrichment at grain boundaries, inclusions, precipitates, and connected dislocation cells, and other microstructural heterogeneities	Intergranular or intragranular cracking and interface weakening	Control grain-boundary segregation, inclusions, cell networks, precipitate populations, and optimize solution and aging treatments; reduce manufacturing defects; and apply AM-specific post-processing where appropriate	Liu M. et al. (2024), Santos Maldonado et al. (2024), Zhang et al. (2019)
Titanium alloys	Hydrogen occupies international sites in the in alpha (α) and beta (β) phases. Its solubility and diffusivity depend strongly on phase constitution, with β phase generally accommodating more hydrogen. Hybrid may form when local solubility limits are exceeded, particularly in α-rich regions	Intergranular cracking, hydride-assisted initiation, local hardening, and severe ductility loss	Limit hydrogen entry, control α/β phase fraction and morphology; reduce defects and residual stress, and optimize heat treatment; and prevent local conditions that promote hydride precipitation	Nguyen et al. (2024), Roudnicka et al. (2024), Zhang et al. (2023)
Aluminum alloys	Hydrogen entry is strongly influenced by the surface oxide film and environmental reactions. Once absorbed, hydrogen can interact with vacancies, dislocations, pores, inclusions, precipitates, producing highly localized accumulated	Blistering, interface damage, and localized fracture near defects or second phases	Reduce porosity, oxide defects, inclusions, and moisture-assisted hydrogen entry; control precipitate and grain-boundary structures; optimize processing and heat treatment; and apply durable surface barriers where appropriate	Jiang et al. (2025), Papantoniou et al. (2024)

The listed responses are major tendencies. Actual susceptibility depends on composition, processing, hydrogen source, concentration, temperature, pressure, stress state, and exposure duration; with BCC: body-centered cubic, and FCC: faced centered cubic.

Hydrogen traps have conditional effects. Conditions such as temperature, microstructure, local hydrogen concentration, reversibility, binding energy, spatial distribution, and mechanical loading determine whether they are useful or deleterious.

Multiphase steels illustrate the importance of phase morphology. For example, compared to ferrite and martensite, austenite is more hydrogen-soluble and has a lower hydrogen diffusivity. Hydrogen mobility can be temporarily reduced by retained austenite, but hydrogen can be released near highly stressed regions when unstable austenite transforms into martensite during deformation. In this regard, improving the hydrogen resistance of 2 GPa direct-quenched and partitioned steels was demonstrated by Pallaspuro et al. (2024) by increasing the thickness of film-like retained austenite.

It is worth noting that, in the BCC phase, aluminum alloying decreased hydrogen attraction and increased hydrogen partitioning toward retained austenite. Evidently, the overall volume of retained austenite is less relevant than its stability, chemistry, and shape. Similarly, the effectiveness of precipitates in increasing hydrogen resistance through the trapping of diffusible hydrogen is size- and location-dependent. Ferritic steels with coherent vanadium carbides maintained a good mix of hydrogen resistance, ductility, and strength, according to Boot et al. (2025b). Despite this, the embrittlement increased from about 15% to 20%–37% when using semi-coherent titanium carbides that were up to 100 nm in size. Hydrogen buildup surrounding grain-boundary precipitates and misfit dislocations facilitated intergranular cracking. As a result, precipitate engineering must prevent hydrogen from accumulating at interfaces that are mechanically weak.

Moreover, manufacturing routes also create distinct hydrogen transport networks. Additively manufactured alloys often contain cellular dislocation structures, residual stresses, segregation zones, pores, and non-equilibrium phases. Santos Maldonado et al. (2024) discovered that the dense cellular dislocation network in the additively manufactured material accelerated hydrogen penetration and changed the fracture mode from intergranular to intragranular cracking. Dislocations acted as both hydrogen traps and rapid diffusion pathways. Therefore, this finding shows that a high trap density does not necessarily improve hydrogen resistance when the traps form connected pathways through the material. Because the same feature may either create connected pathways for localized damage or trap hydrogen beneficially, it remains challenging to establish universal microstructural design principles.

### 3.2 Mechanical loading and surface condition

Hydrogen-assisted failure is highly time-dependent. In particular, at slow strain rates, hydrogen has sufficient time to diffuse toward crack tips, dislocation structures, and regions of high hydrostatic stress. As an illustration, Williams et al. (2024) observed more severe embrittlement in hydrogen-containing electrodeposited copper at a strain rate of 5 × 10^−7^ s^-1^than at 5 × 10^−4^ s^-1^. The slower test permitted greater hydrogen redistribution during deformation. Similar, time-dependent behavior is commonly observed in steels and other hydrogen-sensitive alloys. Additionally, stress concentrations also control local damage. Notches, weld defects, pores, inclusions, and residual stresses increase stress triaxiality and attract mobile hydrogen. For example, there was a robust connection between cyclic loading, hydrogen concentration, crack orientation, and welding residual stress in the numerical study of X80 pipeline girth welds. For pipes with axial cracks, post-weld heat treatment enhanced the anticipated fatigue life by 83%, and for those with circumferential cracks, it was 19%. Indeed, the anticipated fatigue life was 77% shorter when the crack reached the outer pipe surface when the hydrogen fraction was increased from 1.5% to 10% (Xue et al., 2024).

Moreover, cyclic loading can be particularly damaging because repeated plastic deformation continuously creates dislocations and exposes fresh crack-tip surfaces. To illustrate, Okayasu et al. (2024) found that surface treatment changed hydrogen uptake in SUS304 stainless steel by altering roughness, work hardening, internal strain, and strain-induced martensite formation. Although cold-rolled specimens contained relatively high hydrogen concentrations without clear embrittlement during tensile testing, severe degradation occurred during fatigue testing. Accordingly, the authors associated this behavior with hydrogen migration toward the advancing fatigue-crack tip.

Consequently, the surface state influences the entrance of hydrogen and the beginning of cracks. Various processes such as grinding, shot peening, cold deformation, welding, and machining can cause residual stresses to increase, martensitic transformation to occur, and dislocation-rich layers to form. While these alterations could lead to better hydrogen absorption, the controlled surface treatments could decrease fracture opening through compressive residual stresses. Here, new tests on hydrogen-charged 316L stainless steel showed that specimens that were shot-peened had slower fatigue-crack growth rates and fewer hydrogen-induced cracks in the compressive residual-stress layer (Park and Kim, 2025). Ultimately, the overall response depends on the balance between reduced tensile stress and crack opening and increased hydrogen entry and trapping.

On the other hand, compressive residual stress, which normally provides protection, can lose some of its effectiveness when exposed to hydrogen. For instance, in their study, Tien et al. demonstrated that electrolytic hydrogen charging could increase surface hardness while permanently relieving some of the compressive stress caused by shot peening. Hydrogen charging did not affect the ductile fracture behavior of shot-peened specimens as much as unpeened specimens (Tien et al., 2025a; Tien et al., 2025b). Accordingly, to enhance surface treatments, it is necessary to consider their cumulative impacts on residual stress, dislocation density, roughness, phase transition, and hydrogen redistribution, rather than limiting oneself to surface hardness alone. Yet, the interacting effects of service stress, surface evolution, hydrogen redistribution, and long-term cyclic exposure cannot be adequately replicated by the majority of laboratory loading techniques.

### 3.3 Temperature, hydrogen pressure, and corrosive environment

Temperature affects hydrogen adsorption, diffusion, trapping, and desorption simultaneously. Generally, an increase in temperature accelerates lattice diffusion, but it can also release hydrogen from reversible traps. Consequently, the highest embrittlement susceptibility often occurs within a material-specific temperature range rather than increasing continuously with temperature. To illustrate, Xing et al. (2024) identified the most severe embrittlement temperatures for X70 pipeline steel as 293 K under a charging current of 10 mA cm^-2^ and 283 K under 20 mA cm^-2^. Notably, a distinct critical temperature was determined by recent fracture-toughness experiments conducted on X52, X60, and X70 steels, indicating that the impact of temperature is dependent on the steel grade and environmental condition (Gyaabeng et al., 2026). However, at a higher charging current, the authors did not observe a distinct critical temperature. Thus, this demonstrates that temperature and hydrogen-entry rate interact in controlling fracture.

Similarly, hydrogen pressure also influences the hydrogen concentration available at the metal surface. For instance, Gao et al. (2024) studied X80 pipeline steel between minus 15 °C and 60 °C under hydrogen pressures of 1, 4, and 8 MPa. At 4 and 8 MPa, the calculated hydrogen concentration increased with temperature. Conversely, at 1 MPa, it initially decreased and then increased. As a result, this outcome shows that hydrogen-pressure effects cannot be separated from temperature, trap density, and trap-binding energy. Material qualification should therefore cover the pressure and temperature combinations expected during operation.

Likewise, corrosive environments introduce additional complexity because they generate hydrogen electrochemically while damaging protective surface films. Moisture, carbon dioxide (CO_2_), hydrogen sulfide (H_2_S), oxygen (O_2_), chloride ions, and other impurities can alter corrosion-product formation and hydrogen-entry kinetics, and fatigue-crack growth. For example, FeCO_3_ corrosion product scales reduced gaseous hydrogen adsorption and permeation by forming a surface barrier on pipeline steels (Xu et al., 2024). On the other hand, CO_2_ accelerated hydrogen-assisted fatigue-crack growth in pipeline steels. However, its stimulating effect is less strong at 10 MPa compared to 0.4 MPa because CO_2_ adsorption decreases with increasing hydrogen pressure (Shang et al., 2024). Another study by Shang et al., 2024, showed that CO_2_ enhanced hydrogen-assisted fatigue damage in L360 steel, while O_2_ prevented and partially mitigated this impact (Zhou et al., 2024b). In addition, Wang et al. (2025) which examined X80 steel under an 8 MPa hydrogen-blended natural-gas environment containing corrosion-inducing impurities. Increasing the hydrogen fraction reduced the number and size of ductile dimples and increased quasi-cleavage features. The measured elongation decreased to 14.3%, below the 18% requirement cited by the authors for API 5L X80 steel. Thus, gaseous contaminants and corrosion byproducts can interact with the steel surface and, depending on their composition, coverage, and stability, either increase or decrease hydrogen entry.

Overall, it follows that the expected combinations of mechanical stresses, temperature, impurity composition, moisture, corrosion product condition, and total gas pressure should be replicated during material certification. Otherwise, when the factors of hydrogen uptake, fracture toughness, ductility, and fatigue-crack propagation are examined separately, the combined effects of these factors can be overlooked. However, there is a lack of comprehensive datasets that address practical variables such as temperature, pressure, contaminants, moisture, and loading, which makes it difficult to make accurate predictions about service life.

### 3.4 Differences among major engineering alloys

To begin, the fast diffusion of hydrogen through the BCC lattice makes high-strength ferritic and martensitic steels extremely susceptible. Their carbide interfaces, prior-austenite grain boundaries, residual stresses, and high dislocation densities make them ideal places for hydrogen to accumulate. Hydrogen can be delayed in materials with smaller grains and higher dislocation density, but it can be concentrated near weak interfaces in materials with coarse or poorly distributed carbides (Boot et al., 2025b; Islam et al., 2025).

Likewise, alloys based on nickel also have good corrosion resistance and mechanical strength, but they can still crack when exposed to hydrogen. Segregation zones, dislocation cells, grain-boundary precipitates, and inclusions can all regulate the start of cracks. Hydrogen reactions in additively manufactured materials may vary from those in wrought materials (Santos Maldonado et al., 2024) due to the possibility of additional microstructural features. Even so, the primary dangers associated with titanium and aluminum alloys are different. Titanium alloys, particularly those with a metastable or highly strained microstructure, can form brittle hydrides when exposed to hydrogen concentrations that exceed their local solubility limit. Due to the formation of flaws, high residual stress, and tiny metastable microstructures, laser powder bed fusion might enhance susceptibility.

To illustrate, under the same electrochemical charging circumstances, Roudnicka et al. (2024) discovered that as-built laser powder bed fused Ti-Al-6-V-4 exhibited a higher susceptibility and absorbed more hydrogen than annealed and conventionally produced ones. Hydrogen can collect in vacancies, precipitates, grain boundaries, pores, and oxide surfaces, but hydrogen diffusivity is low in aluminum. Similarly, recent research conducted by Papantoniou et al. (2024) revealed that cathodic hydrogen charging diminished the AA6082-T6 aluminum alloy’s mechanical characteristics.

Overall, hydrogen compatibility must be examined for each combination of alloy, processing history, surface condition, loading mode, pressure, temperature, and ambient chemistry. Although a material may hold up well when exposed to gas for brief periods, it may degrade when subjected to gradual loading, cyclic operation, electrochemical charging, or corrosive contaminants. Table 1 lists the hydrogen reactivity of common alloys along with the relative importance of their mitigation strategies.

Thus, it is still difficult to directly compare different alloy because variations in charging techniques, exposure settings, testing methodologies, and susceptibility measurements, consistent performance ranking.

### 3.5 Emerging and advanced material classes for hydrogen service

Emerging material classes can manage hydrogen entrance, trapping, transport, and fracture, although standard steels and Ni-, Ti-, and Al-based alloys are still vital for hydrogen infrastructure. Furthermore, this part examines advanced high-strength steels, high- and medium-entropy alloys, additively created alloys, and other promising options. Their composition- and processing-based resistance mechanisms and expanding importance to hydrogen transport, storage, compression, and conversion systems made these classes suitable. Their performance relies on microstructure, processing history, hydrogen pressure, temperature, loading mode, and exposure length. None is hydrogen compatible overall. To address the growing number of materials proposed for hydrogen-energy systems, Table 4 examines four new material classes based on representative compositions, future uses, hydrogen-related benefits, and current limits.

**TABLE 4:** Comparison of advanced and emerging material classes for hydrogen energy system.

Material class	Representative material	Hydrogen-energy relevance and potential applications	Potential advantages in hydrogen service	Limitation and research need	Refs
Advanced high-strength steels (AHSS)	These steels are press-hardened, quenched, and partitioned, medium-Mn, dual-phase, and TRIP.	Lightweight pressure-containing components, hydrogen-transport structures, and automobile body-in-white components	Resistance may be improved and hydrogen transit restricted by having a high strength-to-weight ratio, a film-like stable maintained austenite, and regulated trapping sites	Martensite composition, carbide distribution, retained-austenite stability, and strength determine susceptibility. Strain-induced austenite transformation can promote hydrogen redistribution and local cracking, while hydrogen charging and loading rate affect the response	Liu et al. (2017), Pallaspuro et al. (2024), Sun et al. (2020)
High-entropy alloys (HEAs)	Transition metal alloys with FCC and multiple phases, especially those including CoCrFeMnNi and systems based on CoCrNi	Potential components of hydrogen energy systems that are safety-critical, including materials for the production, storage, and transportation of hydrogen	Combining good hydrogen resistance with high ductility and strength is possible in some formulations. Resistance could be caused by factors such as low hydrogen diffusivity, lattice distortion, deformation twinning, and interactions between grain boundaries or twin boundaries	The conditions of hydrogen exposure, grain size, processing history, phase constitution, and composition all have a significant impact on performance. There is less consistent and smaller evidence available compared to ordinary steels	Ding et al. (2024), Luo et al. (2017)
Additively manufactured (AM) alloys	Materials that can be printed include stainless steel (AM316L), inconel 718, titanium, aluminum, and other metal alloys	Manufacturing intricate yet lightweight parts for use in energy systems that store or transport hydrogen, such as fuel cells, electrolyzers, and related technologies	Potential hydrogen-trapping locations include microstructural control and geometrical flexibility, as well as cellular dislocation structures and phase boundaries. A number of AM alloys have reduced hydrogen embrittlement sensitivity compared to their traditionally made equivalents	The possibility of localized hydrogen accumulation or movement can also be enhanced by cellular structures, porosity, anisotropic grains, residual stresses, and nonequilibrium phases. Specific qualification for the alloy is required for build orientation, processing parameters, flaws, and heat treatment after construction	Behvar et al. (2024)
Other emerging candidates	Metallic glasses, such as amorphous alloys based on Zr and Ni-Nb, and metal-matrix composites, are potential areas for further study	Possible materials for hydrogen storage, membranes that allow hydrogen to permeate, components for fuel cells, and specific uses for hydrogen contact	Metallic glasses have a lack of the usual diffusion channels and long-range crystallographic flaws. Substantially observable ductility is maintained below a critical hydrogen concentration in certain metalloid-free compositions that may absorb large amounts of hydrogen	The composition has a significant impact on the hydrogen response. Even with low quantities of hydrogen, some glasses containing metalloids, such as those based on iron or nickel, become brittle. There is still a lack of evidence on metal-matrix composites to draw any broad conclusions about their hydrogen compatibility	Jayalakshmi et al. (2012)

The growing importance of hydrogen in many applications necessitates the selection of these classes since they reflect different approaches to material design and manufacture. Their performance is nevertheless dictated by the conflict between hydrogen transport, trapping, local accumulation, microstructural evolution, and mechanically induced damage, even though they are different.

## 4 Advanced characterization and predictive approaches

Methods that can determine the amount of hydrogen that enters a material, its accumulation sites, its rate of movement, and its effects on deformation and fracture are necessary for a reliable assessment of HE. Due to hydrogen’s mobility, weak analytical signals, and potential escape or redistribution during specimen preparation, no one method can address all of these requirements. Thus, the current state of the art integrates computer modeling, operando observations, spatially resolved microscopy, and measurements of bulk hydrogen (Aksoy et al., 2026; Vukkum et al., 2025; Wang et al., 2024).

### 4.1 Hydrogen quantification and permeation analysis

Thermal desorption spectroscopy (TDS) is widely used because it uses controlled heating to monitor the release of hydrogen, which in turn quantifies the absorbed hydrogen and shows how strong different trap populations are. On the other hand, factors including heating rate, specimen size, diffusion distance, and overlapping trap populations determine the peak position. Moreover, new research shows that grinding, storage duration, and sample thickness are the three parameters that can most dramatically alter the TDS spectrum. As an illustration, a combination of TDS and microscopy revealed that the particle-matrix interfaces were the most distinctive feature of TiN and TiC precipitates (Lyu et al., 2025; Silva et al., 2024).

In addition, supplemental data on hydrogen entry and transport can be obtained by electrochemical permeation testing. Hydrogen is delivered to one surface, and the time it takes for it to flow through a metallic membrane and emerge at the other surface is measured. Consequently, the effective diffusivity, steady-state flow, apparent trap density, and subsurface hydrogen concentration can all be determined using permeability curves. For instance, this approach was carried out by Islam et al. (2025) on pipeline steels made at various times. So, the steady-state permeation current was 50% higher, and the effective diffusivity was 97% higher in vintage steel compared to modern steel. The discrepancies were associated with dislocation density, grain size, and pearlite content, demonstrating that permeation tests can differentiate transport behavior dependent on microstructure.

On the other hand, data in bulk or average form is provided by TDS and electrochemical permeation. On their own, they are unable to pinpoint which flaws hydrogen occupies. Particularly in BCC steels with high hydrogen mobility, underestimation can occur due to hydrogen loss between charging and testing. For this reason, it is critical to use consistent specimen dimensions, controlled storage temperatures, and rapid transfer when attempting to achieve similar results.

### 4.2 Spatially resolved, *in situ*, and operando characterization

Deformation and fracture caused by hydrogen can be studied directly using electron microscopy. For example, when the mechanism of fracture changes, scanning electron microscopy can detect it. This includes when ductile dimpling becomes quasi-cleavage or intergranular cracking. In the same way, the evolution of defects caused by hydrogen, nanoscale precipitates, dislocation structures, and hydrides can all be seen through transmission electron microscopy. Furthermore, hydrogen charging, in conjunction with *in situ* mechanical testing, captures slip localization, crack initiation, and crack propagation while hydrogen interacts with the deforming microstructure, increasing the mechanistic value.

Additionally, with atomic-level spatial resolution, atom probe tomography (APT) provides three-dimensional compositional mapping. Dislocations, grain boundaries, precipitates, and phase interfaces can all be detected using this method for hydrogen or deuterium segregation. Yet, quantification can be made more difficult by residual hydrogen in the analysis chamber, hydrogen added when preparing the specimen, and hydrogen lost during transfer. To illustrate, for X65 pipeline steel, Wang et al. (2025) thoroughly examined APT processes. Electropolishing introduced hydrogen, and larger negative electrochemical potentials improved the measured hydrogen concentration; they also showed that charged deuterium and hydrogen could be measured with statistical confidence following ambient transfer. As a result, the findings demonstrate the need for meticulously controlling the preparation and charging parameters when comparing APT datasets.

Also, reducing hydrogen redistribution and improving preservation of the original distribution can be achieved through cryogenic transfer. For instance, a quasi-in situ cryogenic-transfer APT method was devised by Vukkum et al. (2025) to study deuterium out-diffusion from an austenitic FeCrNi alloy. The oxide layer acted as a permeation barrier, slowing deuterium release, according to repeated studies conducted before and after controlled heat treatments. Research also showed that surface coatings, transfer temperature, pulse mode, and exposure history are important factors to consider when analyzing APT data.

More recently, operando methods have begun to overcome the limitations of *postmortem* analysis. As an illustration, to map hydrogen infiltration in martensitic steel in real-time, Aksoy et al. (2026) utilized high-energy X-ray diffraction microscopy. Density functional theory calibration was used to transform hydrogen-induced lattice strain into local hydrogen concentration. Consequently, the procedure differentiated between trap-controlled diffusion and fast lattice transport, and it generated spatial and temporal maps of hydrogen transport throughout the specimen. Nonetheless to martensite, which allowed for deeper and quicker penetration, precipitation-rich steel restricted hydrogen close to the surface. As the traps were filled up more and more during charging, the effective diffusivity shifted accordingly. This finding calls into question the conventional wisdom regarding hydrogen diffusivity as an immutable material constant.

Overall, understanding the relationship between hydrogen content, microstructural evolution, and mechanical response is made easier by operando characterization. Nevertheless, specialist facilities and intricate data interpretation are necessary for such procedures. Therefore, to get a more accurate multiscale description than any one method could provide, researchers should operando x-ray or neutron techniques with TDS, permeation testing, electron microscopy, and APT. Despite this, operando approaches are not yet widely used or cross-validated due to their high cost, difficulty in interpretation, and restricted accessibility.

### 4.3 Atomistic and continuum modelling

It is possible to determine the energies of hydrogen solution energies, migration barriers, trap-binding energies, interfacial cohesion, and interactions with alloying elements using Density Functional Theory (DFT). The basic knowledge that these computations give is hard to get empirically. However, complicated grain boundaries, dislocation networks, and propagating cracks cannot be directly simulated due to their limited accessible length and time scales. Conversely, by simulating hydrogen’s interactions with flaws as they undergo deformation, molecular dynamics increases the accessible system size. However, the accuracy is highly dependent on the interatomic potential that is employed. Hydrogen behavior in diverse defect environments cannot be described by conventional empirical potentials, even though they can reproduce specified material properties. Therefore, one way to get near-DFT precision with less processing expense is by using machine-learning interatomic potentials.

At the engineering scale, diffusion, crystal-plasticity, and phase-field fracture models describe complementary hydrogen-assisted degradation processes. Lattice diffusion and reversible or irreversible trapping can be included in diffusion models to estimate hydrogen transport and redistribution under concentration, chemical-potential, temperature, and stress gradients. Crystal-plasticity models link microstructural heterogeneity to local hydrogen accumulation by resolving grain-scale deformation, crystallographic slip, dislocation activity, and stress localization. Instead of predetermining the crack direction, phase-field fracture models simulate crack initiation, branching, and propagation. Modern coupled formulations combine hydrogen transport, trapping, plastic deformation, hydrostatic stress, and hydrogen-dependent fracture resistance. Liu H. et al. (2025) created a coupled phase-field framework for hydrogen-assisted fatigue cracking. The model related stress-assisted hydrogen diffusion, fatigue damage, and hydrogen-dependent fracture energy deterioration. It also simulated how local hydrogen accumulation affects crack initiation and propagation, allowing hydrogen exposure and cyclic loading to be studied.

Nevertheless, model predictions are still sensitive to factors including surface hydrogen concentration, binding energy, diffusivity, trap density, and hydrogen-dependent toughness, even with these improvements. Moreover, these numbers can change depending on the experiment. Therefore, multiple independent measurements taken at appropriate pressures, temperatures, and loading rates should be used to calibrate and validate predictive models.

### 4.4 Artificial intelligence and machine learning

Machine learning has two primary applications that can aid in the creation of hydrogen-compatible materials. First, they can accelerate the acceleration of atomistic simulations using machine-learning interatomic potentials. Second, they can identify it can identify relationships among alloy composition, processing conditions, hydrogen exposure, microstructure, and mechanical degradation.

To illustrate, Ito et al. (2025) constructed a machine-learning interatomic potential for the Ni-Mn-H system using active learning. Molecular-dynamics simulations reproduced the experimentally observed nonlinear effect of manganese content on hydrogen diffusivity. Specifically, the analysis showed that manganese produced competing effects through repulsive Mn-H interactions and lattice expansion. The balance between these effects changed with Mn concentration and controlled hydrogen migration. Besides, a later study developed a transferable Fe-H machine-learning potential for general grain boundaries in α-iron. Large-scale simulations indicated that hydrogen segregation could suppress full dislocation emission from several grain boundaries, reducing plastic accommodation and increasing the likelihood of interfacial fracture. Thus, the study demonstrated how machine-learning potentials can connect atomic hydrogen segregation with grain-boundary deformation at scales that are difficult to reach using DFT alone.

In addition, machine learning can also assist experimental interpretation. To illustrate, Guo et al. (2025) used a transfer-learning ResNet50 model to identify hydrogen-affected regions on fracture surfaces of additively manufactured 18Ni300 maraging steel. The model was then used to estimate hydrogen penetration depth and diffusivity from fractographic images. This approach illustrates the potential of automated image analysis to reduce subjective classification of ductile and brittle fracture features.

It is important to differentiate between the many AI methodologies utilized in hydrogen embrittlement research. According to Zhang S. et al. (2024), atomistic simulations may access hydrogen interactions with defects, grain boundaries, and fracture tips through machine-learning interatomic potentials, which simulate atomic energies and forces. On the other hand, computer-vision models can identify microstructures or surfaces impacted by hydrogen in pictures, while traditional supervised-learning models may forecast ductility loss or fracture toughness using labeled data on composition, processing, and testing (Guo et al., 2025). Unfortunately, without sufficiently big, varied, and independently validated experimental datasets, these models can only learn correlations within their training domains and will not be able to give predictions that are generally accurate (Xu et al., 2023) emphasize the importance of uncertainty assessment and validation against unseen materials and service situations.

## 5 Mitigation and hydrogen compatible materials design

Surface hydrogen formation, internal transport, trapping, stress-assisted buildup, and fracture propagation are all steps that must be controlled in order to mitigate HE. However, rarely can all of these processes be adequately protected by a single measure. Therefore, a well-designed material will have stable microstructures, traps that are dispersed correctly, interfaces that are resistant, surface barriers that are durable, and manufacturing stresses that are controlled.

Managing the entrance, transportation, trapping, and accumulation of hydrogen at areas susceptible to cracks is essential for preventing hydrogen embrittlement. That is why there is not a silver bullet approach; results vary depending on factors including alloy microstructure, production method, loading situation, and service environment. The main mitigation solutions are compared in Table 5 with respect to their benefits, limitations, scalability, and service-related concerns.

**TABLE 5:** Strategies for reducing hydrogen embrittlement with an emphasis on decision-making.

Strategy	Benefits	Limitations/failure modes	Scalability	Service-condition concerns	Refs
Alloy and trap engineering (5.1)	Immobilizes diffusible hydrogen away from fracture-sensitive sites; can be built into bulk alloy design	Overly strong/large or poorly located traps can concentrate hydrogen at grain boundaries; long-term trap stability under cyclic load is largely unverified	High alloying/precipitation routes are compatible with standard steelmaking	Trap stability under cyclic loading and prolonged high-temperature service is not yet established	Liu M. et al. (2024), Ji et al. (2025)
Interface and inclusion engineering (5.2)	Reduces hydrogen segregation at fracture-sensitive boundaries; cuts hydrogen ingress (e.g., ~50% reduction reported for boron/carbon-enriched interfaces)	Requires precise control of segregation chemistry; large/elongated/clustered inclusions remain a risk if not controlled	Moderate achievable via alloy chemistry and thermomechanical processing, but interface chemistry control at industrial scale is challenging	Long-term stability of engineered interfaces under service loading and thermal cycling is not established	Hachet et al. (2025), Shi et al. (2025)
Surface modification and hydrogen barriers (5.3)	Directly reduces hydrogen entry into the structural alloy; several coating/nitride chemistries demonstrated substantial diffusivity reduction	Performance is coating-specific and sensitive to defect density, adhesion and wear; mechanical damage can create local hydrogen accumulation and blistering	Variable some methods (e.g., gas nitriding) are scalable surface-conversion processes; others require deposition equipment	Barrier integrity under pressure cycling, abrasion, corrosion and thermal cycling is largely untested	Albrecht et al. (2025), Sugawara and Kudo (2023)
Heat treatment and manufacturing control (5.4)	Homogenizes microstructure, reduces dislocation density and residual stress, can reduce HE sensitivity to conventionally manufactured levels	Inappropriate treatment can remove beneficial traps, coarsen precipitates, or produce brittle phases	High for wrought products; more variable for additively manufactured/welded components	Optimal treatment windows remain conditional on component geometry, production route and service environment	Liu et al. (2025a), Sridharan et al. (2025), Álvarez et al. (2023)

When it comes to mitigation, the best method is to limit bulk microstructural factors while also engineering interfaces, protecting surfaces, and using the right manufacturing or heat-treatment conditions. Poorly placed, reversible, or saturated traps can enhance local hydrogen accumulation; therefore, reduced hydrogen diffusivity or robust trapping does not always ensure enhanced resistance. Hence, environmental, loading, pressure, temperature, and exposure variables that are reflective of the real world should be used to assess mitigation strategies.

### 5.1 Alloy and hydrogen trap engineering

The resistance of metallic materials to HE is anticipated to be enhanced by ongoing developments in alloy design, microstructural engineering, and surface modification. Intrinsically resistant alloys can be developed by introducing microstructural features that immobilize diffusible hydrogen. An effective trap requires appropriate binding energy and number density and must not create a preferential fracture path. Hydrogen can be released during loading by weak traps or concentrated around grain boundaries and other susceptible interfaces by overly powerful or improperly placed traps.

For example, this idea was shown by Liu M. et al. (2024) in their demonstration of ferritic steel with TiC and mixed Ti and Mo carbide precipitates. The carbide chemistry was changed, and carbon vacancy sites were formed within the precipitates by adding molybdenum. Steel with Ti-Mo carbides kept 1.685 weight ppm hydrogen after 1 hour of room temperature desorption, while TiC steel retained only 0.169 weight ppm. Besides, Ti-Mo carbide steel exhibited reduced hydrogen permeability at the macroscopic level as well. Instead of staying at the carbide/matrix boundary, atomic probe tomography showed that deuterium went into the carbide itself. These results demonstrate that precipitate chemistry, rather than precipitate number alone, can be tailored to increase trapping capacity.

Moreover, both the size of the trap and the coherency of the interface are critical. Ferritic steels with TiC and VC precipitates of varying sizes were compared by Boot et al. (2025b). Under most circumstances, a 15%–20% decrease in ductility due to hydrogen was observed. Hydrogen collected around misfit dislocations and grain boundary precipitates, facilitating intergranular fracture, while steel containing semi-coherent TiC particles as large as 100 nm exhibited 37% embrittlement. On the other hand, under hydrogen charge, steel with coherent VC nanoparticles retained the optimal strength-to-ductility ratio. Thus, uniformly distributed coherent nanoparticles appear more suitable than large, incoherent precipitates near fracture sensitive boundaries. Overall, these studies indicate that trap engineering should consider four linked properties: trap binding energy, number density, spatial distribution, and mechanical compatibility with the surrounding matrix. Increasing hydrogen uptake is beneficial only when the trapped hydrogen remains isolated from crack initiation sites during the intended service period. There has not been enough testing to confirm that constructed traps are stable and effective over the long term when subjected to cyclic loads and high temperatures.

### 5.2 Interface and inclusion engineering

Grain boundaries, martensite boundaries, phase interfaces, and inclusion interfaces often serve as hydrogen accumulation and crack propagation sites. Hence, their chemical modification provides a direct strategy for limiting hydrogen segregation and maintaining interfacial cohesion. For instance, Hachet et al. (2025) used atomistic calculations and experiments to design boron and carbon-enriched interfaces in low-carbon martensitic steel. Boron segregated mainly to prior austenite grain boundaries, while low-temperature tempering promoted carbon segregation at martensitic interfaces and reduced internal stresses. Combining boron addition with tempering decreased the saturated hydrogen concentration from about 280 atomic ppm in untreated steel to 120 atomic ppm. Additionally, it reduced prior-austenite grain-boundary cracking to approximately 10%. Likewise, the overall amount of hydrogen that leaked out was cut in half. Collectively, the outcomes demonstrate that interstitial solutes have the ability to deflect hydrogen away from susceptible interfaces, enhance boundary cohesiveness, and lessen stress caused by quenching. Similarly, pipeline steels have an additional option with inclusion engineering. The process of hydrogen-induced cracking can be initiated by inclusions that are large, elongated, crowded, or have weak bonds. However, fine and uniformly dispersed inclusions can provide distributed trapping sites and support beneficial microstructure formation. By way of example, Peng et al. (2024) modified pipeline steel using different Ti/Mg ratios. Steel produced with a Ti/Mg ratio of 4:1 contained small, spherical, and uniformly dispersed multicomponent inclusions and showed the best resistance to hydrogen-induced cracking. It also had the lowest effective hydrogen diffusion coefficient and the highest estimated trap density, reaching 5.24 × 10^19^ m^-3^. Nevertheless, inclusion engineering must still avoid creating large oxide or sulfide clusters. Besides, the objective is to refine inclusion size, control shape and chemistry, prevent chain-like distributions, and maintain strong bonding between the inclusion and surrounding matrix. Still, a significant challenge in manufacturing is continuously managing the interface chemistry and inclusion distribution at the industrial scale.

### 5.3 Surface modification and hydrogen barriers

Surface barriers can reduce the quantity of hydrogen reaching the structural alloy. Candidate systems include metallic coatings, nitrides, oxides, carbides, carbon-based layers, polymers, and composite coatings. Their effectiveness depends on hydrogen solubility and diffusivity, coating density, adhesion, thickness, corrosion stability, and resistance to cracking during mechanical or thermal cycling. As an example, Zhou et al. (2024a) electrodeposited WS_2_/Ni composite coatings on X70 pipeline steel. The coatings reduced hydrogen diffusion and permeation while improving mechanical resistance under hydrogen charging. However, performance depended strongly on WS_2_ concentration. Specifically, a concentration of 5 g L^-1^) produced the most effective barrier, while excessive WS_2_ caused particle agglomeration and reduced protection. Overall, the study demonstrates that adding a two-dimensional barrier phase can increase diffusion path tortuosity, but only when the particles remain uniformly distributed within a dense coating.

Similarly, gas nitriding also provides a potentially scalable barrier treatment. Albrecht et al. (2025) produced a γ′ Fe_4_N surface layer on iron and evaluated it using hydrogen permeation testing, atom probe tomography, and density functional theory. Notably, the nitride layer reduced hydrogen diffusion by approximately 20 times at room temperature. Due to the strong migratory barriers and energetically unfavorable hydrogen incorporation, the nitride structure produced the barrier effect. Moreover, surface conversion, rather than material deposition, generates the protective layer directly, making this method appealing. However, it is especially important to pay attention to coating performance when mechanical contact is present. This can be seen in current experimental investigations developed by Shin and Kim (2024), which examined diamond-like carbon coatings with a chromium interlayer on 316L stainless steel for hydrogen valve applications. Hydrogen charging increased coating roughness by as much as 3.8 times and produced a delamination ratio of about 58% at the highest charging current. Consequently, hydrogen entered through surface defects, accumulated at the coating and interlayer interface, and promoted blistering and separation. Furthermore, the adhesion load associated with complete coating failure decreased by 2.0 N. Collectively, these findings show that a coating with high intrinsic barrier properties may still fail if it contains pores, develops interfacial hydrogen accumulation, or experiences repeated wear.

Looking ahead, future barriers should therefore use multilayer or compositionally graded structures that block connected defects and reduce stress differences between the coating and substrate. In addition, long-duration testing should also include pressure cycling, abrasion, corrosion, temperature variation, and local coating damage. There has not been sufficient evidence that the majority of the suggested barriers can withstand pressure cycling, local damage, corrosion, and wear and tear.

### 5.4 Heat treatment and manufacturing control

Heat treatment can alter dislocation density, phase stability, residual stress, precipitate distribution, grain boundary chemistry, and hydrogen trapping. These changes may reduce hydrogen susceptibility, but an inappropriate treatment can remove beneficial traps, coarsen precipitates, or produce brittle phases. To clarify, Liu Q. et al. (2025) investigated directed energy deposited 316L stainless steel subjected to heat treatments at 600, 800, and 1200 °C. Increasing annealing temperature progressively removed cellular subgrain structures, reduced dislocation density, promoted recrystallization, and produced a more homogeneous strain distribution. Notably, the 1200 °C treatment reduced HE sensitivity to a level similar to conventionally manufactured 316L. Despite this, in untreated specimens, strain localized at fewer sites and developed into wide secondary cracks. Meanwhile, heat-treated specimens developed a larger number of more evenly distributed deformation regions, which delayed critical localization.

Moreover, components that are welded or made via additive manufacturing require extra attention to manufacturing control. Regions of hard heat-affected residual tensile stress, segregation, and localized phase changes can all be produced by welding. Similarly, pores, melt pool borders, anisotropic grains, dislocation cell networks, and significant residual stresses are all possible byproducts of additive manufacturing. Therefore, the ideal post-manufacturing treatments would lessen detrimental stress and microstructure instability while maintaining mechanical strength and corrosion resistance. Optimal heat treatment windows are still quite conditional on service conditions, component shape, production route, and alloy composition.

### 5.5 Integrated design for hydrogen service

Material selection should account for the real exposure to hydrogen, operational pressure, temperature, loading frequency, environmental contaminants, joining method, and needed service life while choosing the material. For instance, improvements to pipeline steels could be achieved through the use of internal barrier coatings, controlled carbide trapping, resistant weld areas, and refined inclusions. Likewise, high fracture toughness, microstructure stability, and resistance to cyclic hydrogen exposure are requirements for pressure vessels. Furthermore, surfaces that are resistant to wear and hydrogen permeation are necessary for valves and compressors. Corrosion, electrochemical hydrogen production, and temperature gradients are additional stresses that electrolyzer and fuel cell components must withstand.

Consequently, an integrated design approach is the most dependable option. First, alloys-based traps should be stable and mechanically compatible. Second, heat treatment should alleviate residual stresses and shield delicate contacts. Third, limiting hydrogen penetration without adding fragile or poorly connected layers is the goal of surface engineering. Lastly, it is important that qualification testing mimics the actual service conditions in terms of temperature, environment, loading, and pressure. On the other hand, validated frameworks that integrate alloy design, surface protection, production history, and service-specific certification remain unavailable.

## 6 Conclusion

HE remains a major barrier to the safe and sustainable use of metallic materials in hydrogen generation, storage, transport, and utilization systems. Hydrogen-assisted degradation involves surface reactions, dissociation, absorption, diffusion, trapping, and stress-driven accumulation. Grain boundaries, dislocations, vacancies, inclusions, precipitates, phase interfaces, residual stresses, and crystal structure strongly affect these processes. Thus, no single mechanism governs hydrogen-assisted failure. Furthermore, hydrogen-enhanced localized plasticity, hydrogen-enhanced decohesion, vacancy-assisted damage, and hydride formation can occur independently or simultaneously depending on material, hydrogen concentration, temperature, pressure, loading rate, and environment.

The nature, density, spatial distribution, and interactions of microstructural features with the surrounding matrix determine whether they operate as reversible or irreversible hydrogen traps, affect hydrogen transport and local accumulation, and either prevent or encourage crack development. Large, incoherent particles and susceptible grain boundaries may promote hydrogen accumulation and intergranular cracking, whereas fine, coherent, homogenously distributed precipitates may reduce effective hydrogen mobility. However, increased trapping or reduced diffusivity does not necessarily improve hydrogen resistance because highly populated traps may produce damaging local hydrogen concentrations. Hydrogen resistance can therefore be improved by optimizing precipitate distribution, austenite stability, interface segregation, inclusion size, carbide chemistry, and residual stresses. In addition, heat treatment enhances phase stability and defect and corrosion-resistant treatments can limit hydrogen entry, but their effectiveness depends on adhesion, defect density, durability, and stability under pressure, temperature, wear, and corrosion. However, simplified laboratory conditions limit the applicability of many mitigation strategies to long-term service. There are more possibilities for advanced manufacturing and hydrogen energy than only traditional steels, Ni-based alloys, Ti alloys, and Al alloys. Emerging material classes are opening up these possibilities. The hydrogen response of additively made alloys, metallic glasses, high-entropy and related medium-entropy alloys, high-strength steels, and other materials is still quite conditional on factors like microstructure, processing route, flaws, and service circumstances. Qualification of these materials is thus process- and application-specific as well as material-specific.

Hydrogen concentration, diffusion, trapping, and localization can now be studied across multiple length scales using thermal desorption spectroscopy, electrochemical permeation, atom probe tomography, electron microscopy, X-ray-based methods, cryogenic transfer, and *in-situ* testing. Nevertheless, study comparisons are difficult due to differences in testing methods, specimen preparation, hydrogen charging conditions, and data interpretation.

Computational modelling and artificial intelligence can further link hydrogen interactions to component failure. In particular, phase-field models, density functional theory, molecular dynamics, and machine-learning potentials improve trap design, interface engineering, and fracture prediction. However, these methodologies require validated experimental data, realistic service conditions, consistent datasets, and uncertainty-aware analysis. Future studies should reproduce service-relevant hydrogen pressure, gas composition, temperature, corrosion, fatigue, welding effects, and cyclic loading. Ultimately, progress will be driven by alloy design, microstructure control, interface engineering, surface protection, advanced manufacturing, multiscale characterization, and predictive modelling. Laboratory findings must be translated into qualities materials and components for safe and durable hydrogen-energy systems.
